# Isoaspartic acid is present at specific sites in myelin basic protein from multiple sclerosis patients: could this represent a trigger for disease onset?

**DOI:** 10.1186/s40478-016-0348-x

**Published:** 2016-08-12

**Authors:** Michael G. Friedrich, Sarah E. Hancock, Mark J. Raftery, Roger J. W. Truscott

**Affiliations:** 1 Illawarra Health and Medical Research Institute, University of Wollongong, Wollongong, NSW 2522 Australia; 2 School of Medicine, University of Wollongong, Wollongong, NSW 2522 Australia; 3 Bioanalytical Mass Spectrometry Facility, University of New South Wales, Sydney, NSW 2052 Australia

## Abstract

Multiple sclerosis (MS) is associated with breakdown of the myelin sheath that coats neurons in the central nervous system. The cause of MS is not known, although the pathogenesis involves destruction of myelin by the immune system. It was the aim of this study to examine the abundant myelin protein, myelin basic protein (MBP), to determine if there are sites of modification that may be characteristic for MS. MBP from the cerebellum was examined from controls and MS patients across the age range using mass spectrometry and amino acid analysis. Amino acid racemization data indicated that myelin basic protein is long-lived and proteomic analysis of MBP showed it to be highly modified. A common modification of MBP was racemization of Asp and this was significantly greater in MS patients. In long-lived proteins, L-Asp and L-Asn can racemize to three other isomers, D-isoAsp, L-isoAsp and D-Asp and this is significant because isoAsp formation in peptides renders them immunogenic.

Proteomic analysis revealed widespread modifications of MBP with two surface regions that are altered in MS. In particular, isoAsp was significantly elevated at these sites in MS patients. The generation of isoAsp could be responsible for eliciting an immune response to modified MBP and therefore be implicated in the etiology of MS.

**Electronic supplementary material:**

The online version of this article (doi:10.1186/s40478-016-0348-x) contains supplementary material, which is available to authorized users.

## Introduction

MS has long been thought to be an autoimmune disease. Injection of brain homogenates into animals results in demyelination resembling that seen in MS [33] and a body of data, including the presence of antibodies to myelin components in MS patients, supports a role for an autoimmune response in the genesis of human MS [32, 42].

MBP accounts for 35 % of myelin protein and is intrinsically unstructured [16] Recently, MBP and other proteins in myelin were shown to be long-lived [35]. Over time, long-lived proteins degrade and this time-dependent deterioration has been well studied in the lens, a tissue with no protein turnover. In lens proteins, age-related post-translational modifications (PTMs) were localized to unstructured regions [19] and racemization, which involves conversion of L- to D-amino acid residues, was found to be the most abundant type of PTM [39]. D-amino acids at some sites were present at levels that exceeded the amount of the precursor L-amino acid [21].

In humans, major proteins from cataract lenses are characterized by specific racemization sites that differ from those in comparable age-matched normal lenses [18, 20, 21]. This finding implies that pathways of protein degradation in the body may possibly determine disease outcome. In long-lived proteins, Asp and Asn residues are particularly sensitive to racemization, undergoing a spontaneous cyclisation reaction that leads to the formation of L-Asp, D-Asp, L-isoAsp and D-isoAsp (see Fig. 2). isoAsp residues are typically present in the highest amounts [13].

Modifications on this scale inevitably have consequences for protein structure and can lead to a functional decline of the protein [34]. Recently it has been shown that the formation of isoAsp can also lead to altered immunogenicity of peptides/proteins, inducing both T and B cell immunity [11]. Thus, like citrulline formation from Arg residues, conversion of Asp/Asn to isoAsp, can potentially induce an immune response to self-antigens [10]. In the current study we examined the hypothesis that since MBP is long-lived and unstructured it, like lens proteins, may also undergo significant covalent alterations. For the reasons outlined above, particular reference was paid to Asp/Asn racemizaton to isoAsp. If specific PTMs such as these were found in the MBP from MS patients, then they could play a role in the etiology of MS by provoking an immune response to the selectively modified myelin.

## Materials and methods

### Tissue samples

Cerebellum samples from control (*n* = 21) and MS patients (*n* = 8) were obtained from the New South Wales Tissue Resource Centre at the University of Sydney with approval from the University of Wollongong ethics committee (Ethics #11/267). MS patients were diagnosed as follows: four with secondary progressive MS (ages 65, 68, 48, 60), two with relapsing remitting MS (ages 70 and 72) and two with primary progressive MS (ages 36 and 62). All MS samples displayed microscopically small lesions with the exception of the 68 year-old patient. Control and MS samples were analysed separately, blind with respect to age and severity of MS. Further details are provided in Additional file 1: Tables S1 and S2.

### Homogenization of human brain tissue

Frozen cerebellum (Gyri of the posterior lobe) from controls (105 +/− 11 mg) and MS patients (115 +/− 13 mg) was pulverized then homogenized as detailed in Norris *et. al.* [30] After homogenization, each sample was transferred to a 5 mL glass tube and centrifuged (1000 *g*, 10 min) at 4 °C. Myelin was enriched by use of a sucrose gradient as described in Larocca and Norton [23]*.*

### Purification of MBP

Myelin basic protein was enriched with modifications to the protocol of Chevalier and Allen [7]. Briefly, the enriched myelin fraction was re-suspended in 50 mM Tris buffer (pH 7.4) and centrifuged (21,000 *g*, 20 min). The supernatant was discarded and the pellet re-extracted. The pellet was re-suspended in 50 mM acetic acid (1 mL) and centrifuged (21,000 *g*, 20 min) and the pellet re-extracted with 50 mM acetic acid. The acetic acid extracts were combined and freeze dried. Greater than 90 % purity of MBP was confirmed by SDS-PAGE (Additional file 1: Figure S2). Since PTMs, such as those associated with aging, can cause major alterations to the properties of proteins, MBP was not separated into isoforms (charge isomers) prior to proteomic analyses [22].

### Amino acid analysis

D-amino acid content of MBP samples was determined by amino acid analysis as described [18]. Three separate runs were carried out for each sample on an Agilent 1100 HPLC system.

### *Capillary LC* mass spectrometry

*Capillary LC* mass spectrometry was undertaken as described [21]. Briefly, MBP (~50 μg) was digested with sequence grade trypsin (1 μg) (Promega) for 16 h at 37 °C. Peptides were desalted and concentrated using a Ziptip (0.6 μL, C18 resin; Millipore) and freeze dried. The lyophilised peptides were re-suspended in formic acid:heptafluorobutyric acid:water (0.1:0.05:98.85). Tandem mass spectra were acquired after LC using a Thermo LTQ Orbitrap as described [21].

Data were searched against the Swiss-Prot database with a range of PTMs using MASCOT (Matrix Science, UK), with enzyme specificity set to trypsin. Peptide tolerance: 1 ppm; fragment tolerance: 0.6 Da with 1 missed cleavage. The following PTMs were listed as variable modifications: deamidation (N,Q,R), oxidation (H,W,M), methylation (R) and phosphorylation (S,T). Routinely greater than 80 % sequence coverage of MBP was observed. To confirm assignments, tandem mass spectrometric fragmentation of each peak was performed and synthetic peptides incorporating the particular modified amino acid (see below for list of commercial standards) were run using the same method to confirm identity by comparison of retention time and MS/MS.

### Data analysis

The doubly charged ions [FFGGDR m/z = 349.66, FFGGD(Cit)GAPK m/z = 526.76, GVDAQGTLSK m/z = 488.25, YLATASTMDHAR m/z = 668.82, TAHYGSLPQK m/z = 551.28 and TAHYGSLPEK m/z = 551.78] and triply charged ions [TQDENPVVHFFK m/z = 487.57 and HRDTGILDSIGR m/z = 447.24] for MBP-derived tryptic peptides were utilised for relative quantification. Their intensities from the extracted ion chromatogram (XIC) were determined using Xcalibur software. Peak areas of each peptide were calculated using a smoothing method [Gaussian, 7 points]. The MS/MS spectrum of each peptide was matched to the XIC, ensuring that the peak areas used corresponded to that of the matched peptide. The percentage of modification was calculated by using: [Modified/(Modified + Non- modified)] × 100.

### Peptide standards

FFGGDR (MBP tryptic peptide 44–49), FFGGDRGAPK and FFGGD(Cit)GAPK (MBP tryptic peptide 44–53), GVDAQGTLSK, (MBP tryptic peptide 143–152), HRDTGILDSIGR (MBP tryptic peptide 32–43), YLATASTMDHAR (MBP tryptic peptide 14–25), TAHYGSLPQK (MBP tryptic peptide 66–75), TAHYGSLPEK (MBP tryptic peptide 66–75) and TQDENPVVHFFK MBP (tryptic peptide 80–91) were synthesized by GLS Biochem (Shanghai, China). FFGGDR, FFGGDRGAPK, FFGGD(Cit)GAPK, HRDTGILDSIGR, YLATASTMDAR, TQDENPVVHFFK and GVDAQGTLSK were synthesized with aspartic acid in four structural isomers, i.e., L-aspartic acid, L-isoaspartic acid, D-aspartic acid, or D-isoaspartic acid. TAHYGSLPQK was synthesized with L- and D- versions of Ser.

### Statistical analysis

Statistical analysis was performed using SPSS Statistics (version 19, IBM Corp. NY, USA) and R (version 3.1.1). Comparison of controls and MS patients was made using a Mann Whitney *U* test with a significance level of *p* = 0.05. Changes to controls with age were analysed by linear regression. Prior to performing linear regression, normality of the dependent variable was assessed by examining the histograms of the standardised residuals and non-normal data were transformed where required.

## Results

### Adult human MBP is extensively racemized

**Fig. 1:**
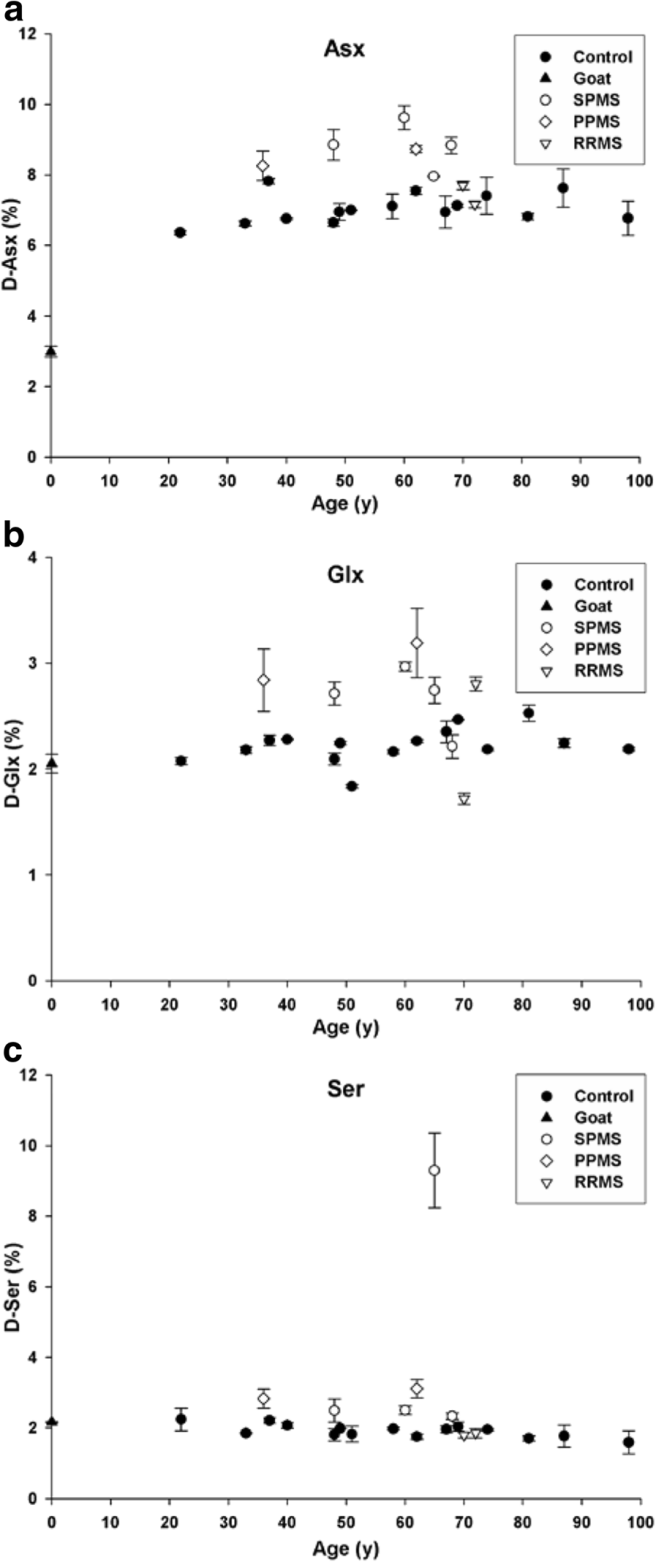
Racemization of **a** aspartic acid and asparagine (Asx), **b** glutamine and glutamic acid (Glx) and **c** serine (Ser) in MBP isolated from controls (●) and multiple sclerosis (MS) patients suffering from SPMS (○) PPMS (◊) and RRMS (▽) as a function of age. Levels of D-Asx (*p* <0.01, Mann Whitney U), D-Glx (*p* = 0.016, Mann Whitney U) and D-Ser (*p* = 0.007, Mann Whitney U) were significantly elevated in MS patients. Age zero corresponds to MBP purified from goat cerebellum (**▼**) and was used as a control for artifactual racemization during hydrolysis. All data are the mean ± SEM of three separate HPLC runs. Racemization in this, and subsequent figures, was expressed as a % $\left(\frac{D}{D+L}\right)$. Controls, *n* = 15; multiple sclerosis patients *n* = 8. In this case, and Figs. 2, 3, 4, 5 statistical analysis was undertaken on combined controls vs combined MS patients

Racemization involves the conversion of an L-amino acid to a D-amino acid and is a defining feature of long-lived proteins e.g. [13, 18]. It significantly affects protein structure and can lead to the formation of epitopes that the body recognizes as being foreign [8]. Initial experiments established the overall degree of racemization of MBP using acid hydrolysis followed by separation of the L- and D-forms of individual amino acids by HPLC [18]. All samples showed substantial racemization, even MBP from a 22 year-old was found to contain a high percentage of D-amino acids (Fig. 1). Indeed the levels of racemization of Asx (i.e. Asn + Asp) (Fig. 1a) were approximately 5 % by age 22 and thus are comparable to those found in human lens proteins [18], which do not to turn over and contain high levels of D-Asp by early adulthood [18]. In lens proteins, there is a rapid age-dependent increase in racemization up to age ~20, after which levels increase much more slowly [9]. This suggests that human MBP is a life-long protein although further testing would be required to confirm this.

Racemization data of MBP from MS patients revealed statistically significant increases in the overall extent of racemization of Asx, Glx and Ser compared to controls (Fig. 1a, b, c). On the basis of the number of Asn/Asp residues present in MBP, on average, approximately one Asp residue in every MBP polypeptide is racemized MS patients. In order to pinpoint the exact sites of modification of MBP, samples were treated with trypsin and the peptides characterized by capillary liquid chromatography tandem mass spectrometry (LC-MS).

### MBP from MS patients differs from controls

To determine the exact sites of modification, MBP was digested with trypsin, which cleaves only at arginine and lysine residues, and the peptide mixture examined by LC–MS. LC enables the separation of racemized forms of each peptide in MBP and tandem MS/MS fragments each peptide giving its sequence and sites of modification. Initial proteomic data showed that the degree of PTM of all MBP samples, both in terms of the number of sites modified, as well as the extent of modification, was considerable. At several sites in MBP there were significant differences in the degree of PTM between controls and MS patients. Individual sites are discussed below with a more comprehensive analysis of all detected PTMs provided in Additional file 1.

### Aspartic acid

**Fig. 2:**
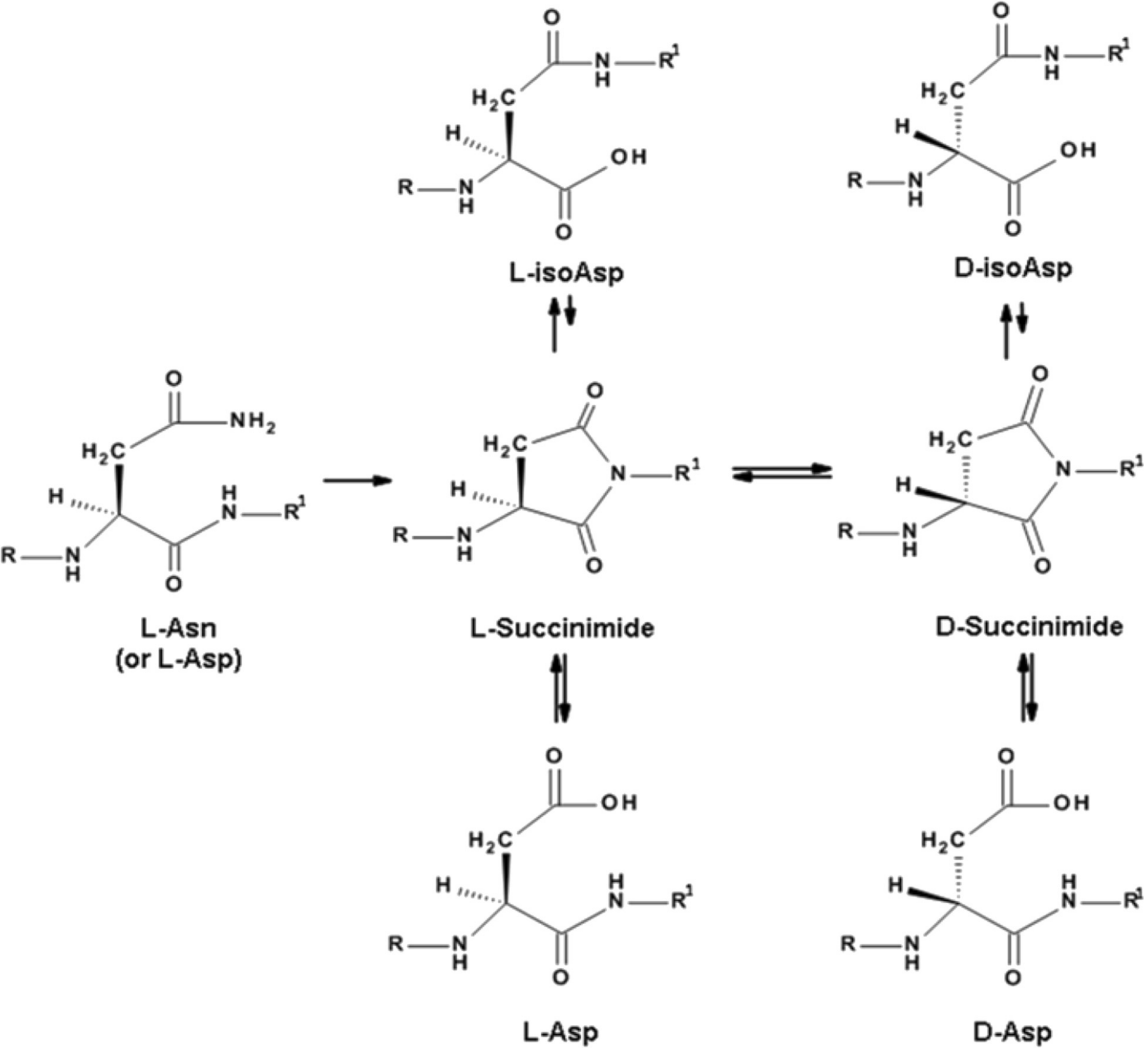
A major source of degradation of long-lived proteins is racemisation. This spontaneous process affects aspartate, asparagine and serine residues in unstructured regions of these proteins. Illustrated is the mechanism responsible for Asp and Asn racemization

**Fig. 3:**
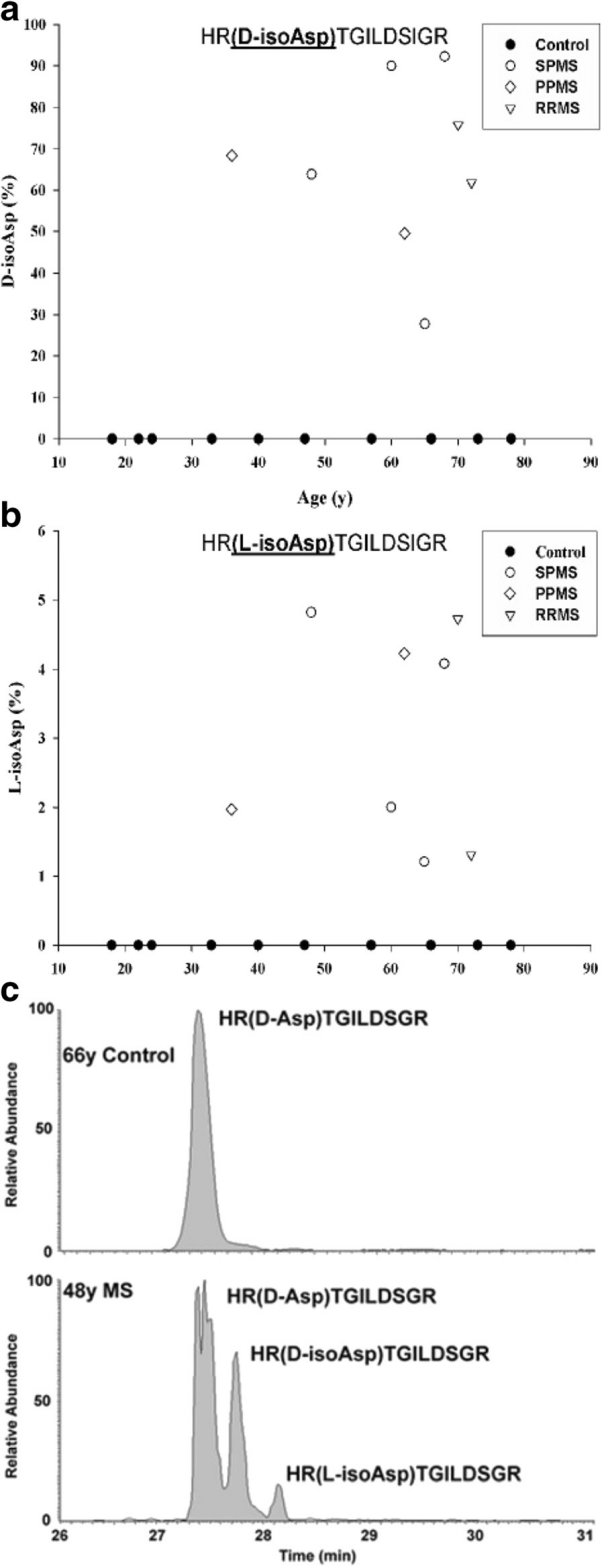
Racemization of Asp34 in MBP from controls (●) and multiple sclerosis (MS) patients suffering from SPMS (○) PPMS (◊) and RRMS (▽). Conversion of L-Asp34 was measured using the tryptic peptide HR*D*TGILDSIGR to (**a**) HR(**D-isoAsp**)TGILDSIGR and (**b**) HR(**L-isoAsp**)TGILDSIGR. Elevated racemization of Asp34 was detected in multiple sclerosis patients for both isoforms: HR(**L-isoAsp**)TGILDSIGR (*p* < 0.001, Mann–Whitney-U) and HR(**D-isoAsp**)TGILDSIGR (*p* = 0.002, Mann–Whitney-U). **c** An example of selected ion chromatographs from the tryptic digest of MBP from a control (66y) and a multiple sclerosis patient (48y). The percentage of modification was determined by the ion intensities of (HR**D**TGILDSIGR)/(HRDTGILDSIGR + HR*D*TGILDSIGR) × 100. Controls, *n* = 10; multiple sclerosis patients *n* = 8

L-Asp and L-Asn residues in long-lived proteins can undergo age-related racemization via an intramolecular condensation involving a succinimide [13]. Hydrolysis of the succinimide produces four structural isomers: L-Asp, D-Asp, L-isoAsp and D-isoAsp [13] (see Fig. 2). The formation of D-Asp, D-isoAsp and L-isoAsp from L-Asp34 in MBP (^32^HR**D**TGILDSIGR^43^) as a function of age is illustrated in Fig. 3. For each of the abnormal Asp isomers there was a significantly greater amount present in MBP from MS patients. Of particular importance, neither D-isoAsp or nor L-isoAsp was detected in the control samples. It should be noted that this peptide contains an internal Arg33 residue that trypsin would normally cleave. We suspected on the basis of previous data [27] that racemization of the adjacent Asp may be responsible. This was tested with four homologous MBP (32–43) peptides. D-Asp and L-isoAsp or D-isoAsp on the C-terminal side of Arg inhibited digestion by tryspin, whereas the L-Asp form was cleaved efficiently.

### Glutamine

**Fig. 4:**
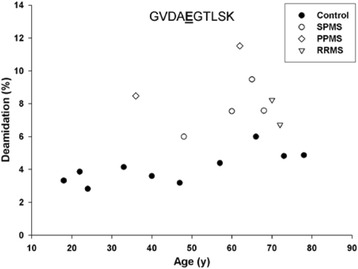
Deamidation of Gln147 in MBP from controls (●) and multiple sclerosis (MS) patients suffering from SPMS (○) PPMS (◊) and RRMS (▽). Deamidation of Gln147 was measured using the tryptic peptide GVDAQGTSK (i.e. GVDAQGTSK to GVDA**E**GTSK). Deamidation was significantly greater in the multiple sclerosis patients (*p* < 0.001, Mann–Whitney-U.). Deamidation increased with age in controls (*R* ^2^ = 0.571, *p* = 0.011)

Deamidation of Gln is another age-related modification of proteins [19]. The deamidation of Gln147 (^143^GVDA**E**GTLSK^152^) as a function of age is shown in Fig. 4. The extent of deamidation of Gln147 increased linearly with age in control MBP, although the values did not exceed 6 %. By contrast, in every case deamidation of Gln147 in MBP from MS patients was greater than 6 % (Fig. 4).

**Fig. 5:**
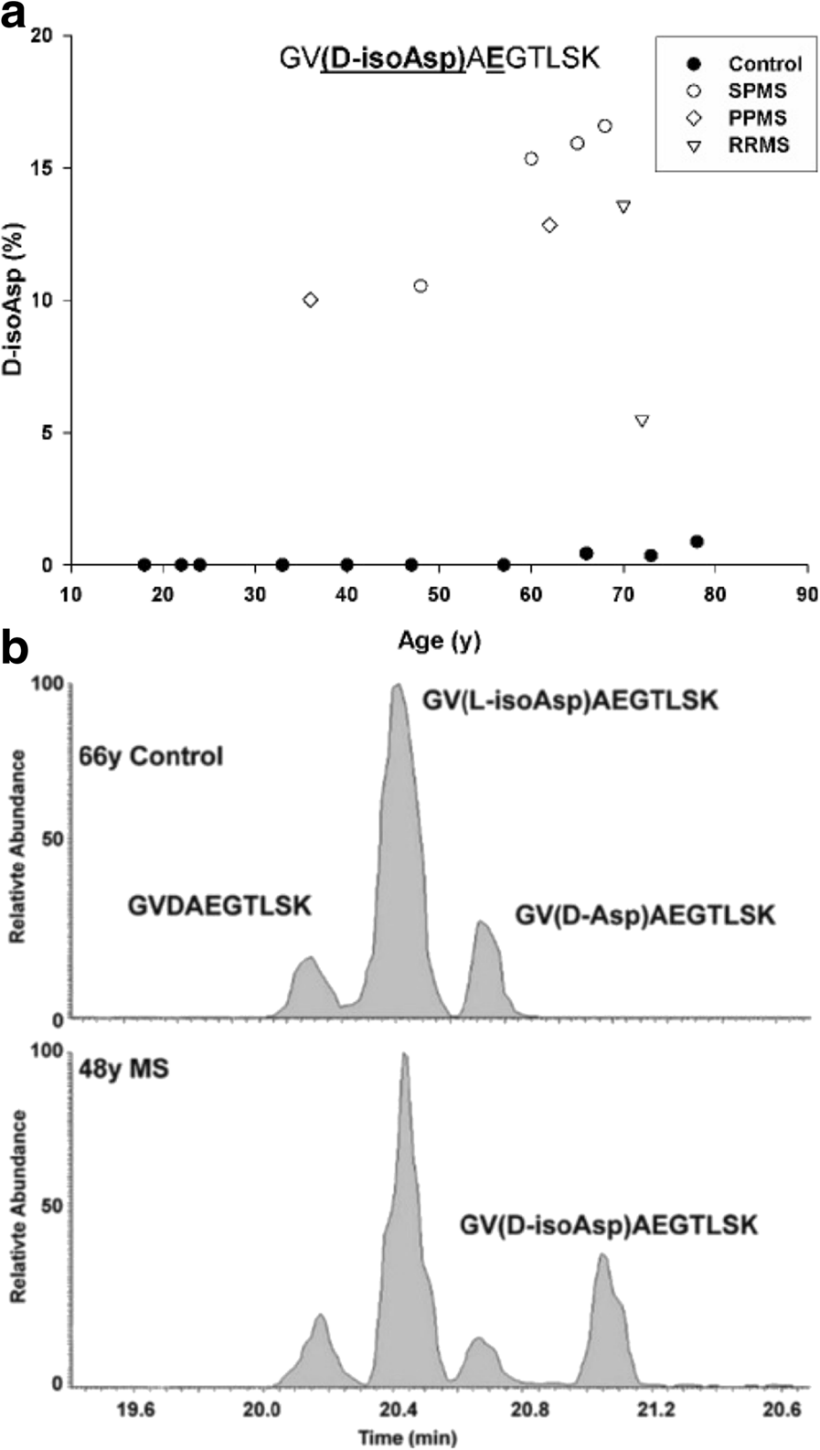
Deamidation of Gln147 coupled with racemization of Asp145 in MBP from controls (●) and multiple sclerosis (MS) patients suffering from SPMS (○) PPMS (◊) and RRMS (▽). **a** Conversion of L-Asp145 to the D-isoAsp form in the deamidated peptide (GVDA**E**GTSK). Racemization of Asp145 to D-isoAsp GV**(D-isoAsp)**A**E**GTSK was significantly greater in multiple sclerosis patients (*p* < 0.001, Mann–Whitney-U). There was no significant difference for the other Asp isomers; GV**(D-Asp)AE**GTSK and GV**(L-isoAsp)AE**GTSK. The percentage of modification was calculated using the ion intensities of (GV**(D-isoAsp)AE**GTSK)/(GV**DAE**GTSK) × 100. **b** Selected ion chromatograph from the tryptic digest of MBP from a control (66y) and an multiple sclerosis patient (48y). Controls *n* = 10; multiple sclerosis patients *n* = 8

Because many amino acid residues in adult MBP were found by proteomic analysis to be modified, in some cases more than one PTM was detected in a single tryptic peptide. This is illustrated in MBP (^143^GVDA**E**GTLSK^152^), where, along with deamidation of Gln, Asp145 was also isomerized. D-isoAsp levels were found to be increased significantly in MS patients compared to controls for the Glu form of this peptide (Fig. 5). The L-isoAsp and D-Asp versions of the Glu version were not significantly different from the controls (data not shown).

### Arginine

Deimination of Arg residues yields citrulline, an amino acid that is not cleaved by trypsin and therefore this PTM leads to missed cleavages during digestion [4]. Fifteen of the 19 Arg sites in MBP showed some degree of conversion to citrulline (Additional file 1: Table S3). Therefore, the relative quantification method used in this study took missed cleavages into account when determining the degree of age-related MBP modification (see Additional file 1: Figure S1).

**Fig. 6:**
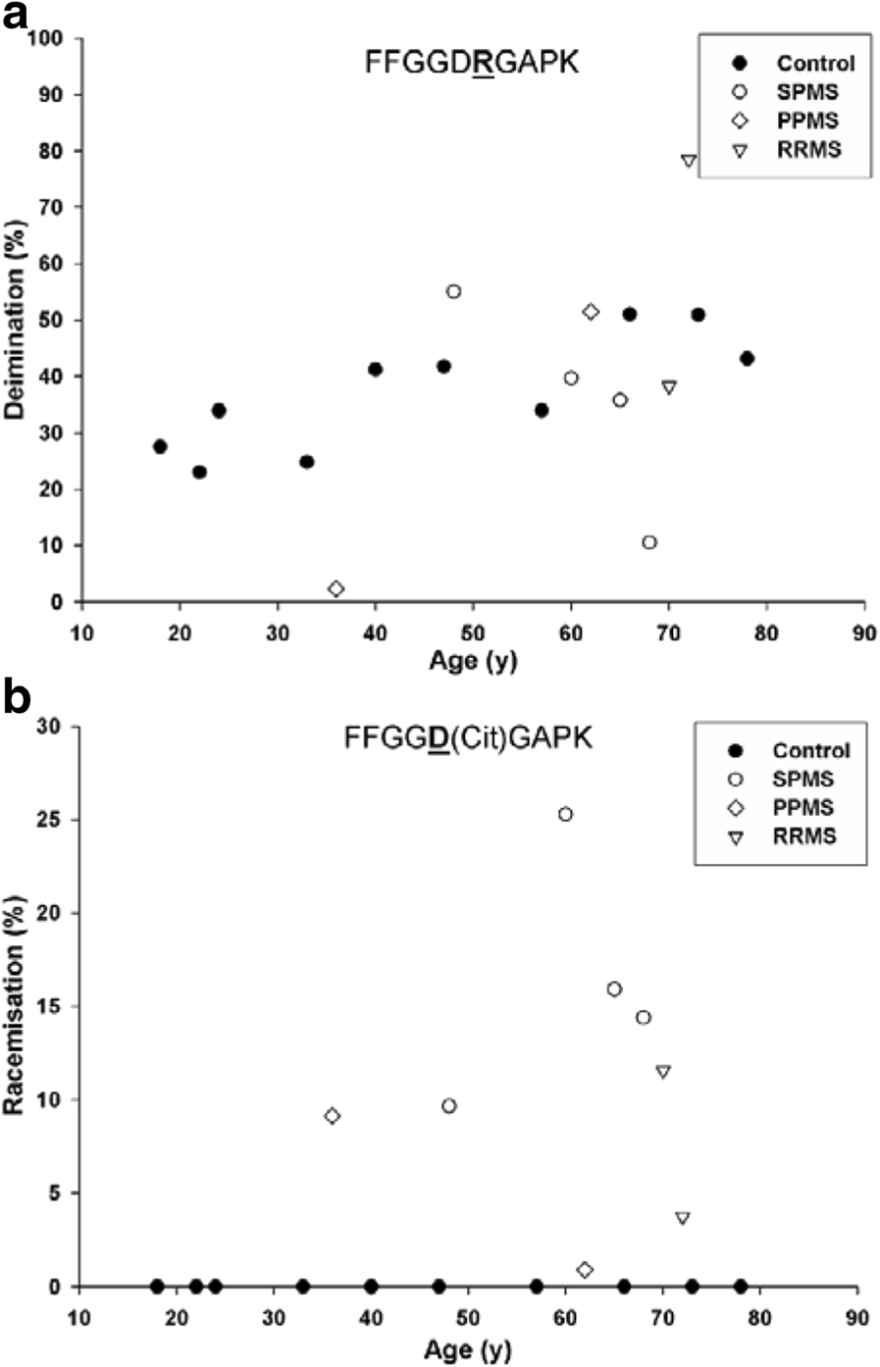
Deimination of Arg49 coupled with racemization of Asp48 in MBP from controls (●) and multiple sclerosis (MS) patients suffering from SPMS (○) PPMS (◊) and RRMS (▽). **a** For the L-Asp version (FFGGD**(Cit)**GAPK), no statistically significant difference was found in the levels of citrulline between controls and multiple sclerosis patients, but linear regression analysis revealed a significant increase in the amount of citrulline with age in control samples (*R* ^2^ = 0.664, *p* = 0.004). **b** Conversion of L-Asp48 to the other Asp isomers in the tryptic peptide deiminated at Arg49 (FFGG**D(Cit)**GAPK). An increase in racemization of Asp48 in the deiminated peptide was seen in multiple sclerosis patients (*p* < 0.001, Mann–Whitney-U). Racemization in this case refers to combined D-Asp, D-isoAsp and L-isoAsp, since the isomers were not separated under these conditions. The percentage of modification was determined by the ion intensities of (FFGG**D(Cit)**GAPK))/(FFGGD**(Cit)**GAPK + FFGG**D(Cit)**GAPK) × 100. Controls *n* = 10; multiple sclerosis patients *n* = 8

Deimination of Arg increased at some sites in an age-dependent manner. This is illustrated for Arg49 (^44^FFGGD**R**GAPK^53^) that appeared to be converted linearly to citrulline (Fig. 6a). This peptide also displayed other MS-specific modifications. In MS samples, deimination of Arg49 was accompanied by isomerisation of Asp48 (^44^FFGG**D**(Cit)GAPK^53^). In MS patients 11 ± 3 % of citrullinated peptides were also racemized at Asp 48. This combination of racemization of Asp48 and deimination of Arg49 was found only in MBP from MS patients (Fig. 6b). Elevated deimination of Arg 65 and Arg 122 was also detected in MBP from MS patients (Additional file 1: Figure S1).

### Interaction between other sites of modification

**Fig. 7:**
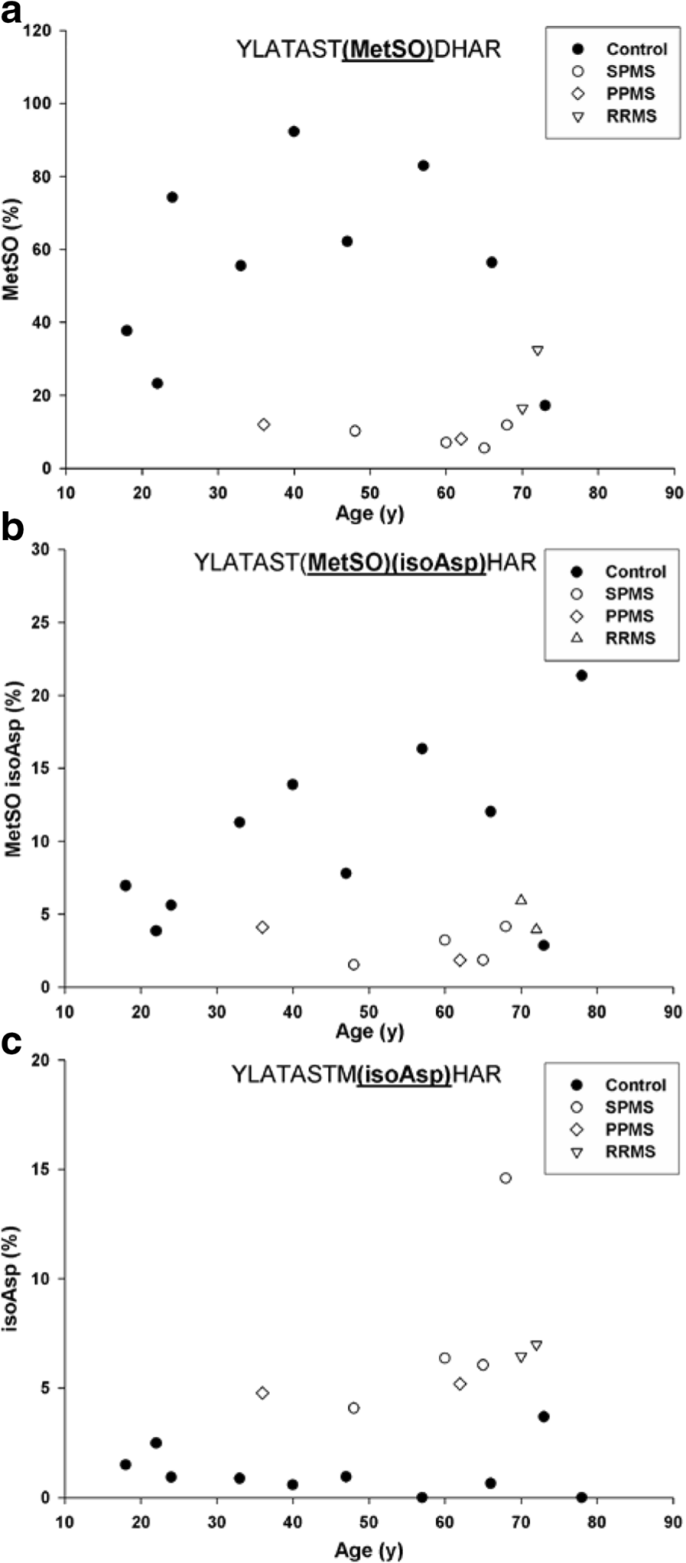
Oxidation of Met22 coupled with racemization of Asp23 in MBP from controls (●) and multiple sclerosis (MS) patients suffering from SPMS (○) PPMS (◊) and RRMS (▽). **a** When all Asp versions of (YLATASTMDHAR) were included, Met oxidation in the controls was significantly greater than in multiple sclerosis patients (*p* < 0.001, Mann–Whitney-U). **b** Oxidation of Met22 and racemization of Asp23 to isoAsp in YLATASTMDHAR. In the Met-oxidized peptide, racemization of Asp23 (YLATAST**(MetSO)(isoAsp)**HAR) was greater in the control samples (*p* = 0.008, Mann–Whitney-U). IsoAsp in this case refers to combined D- and L-isoAsp, since the isomers were not separated under these conditions. **c** Racemization of Asp23 to isoAsp(YLATASTM**(isoAsp)**HAR) in the absence of Met oxidation. Levels of isoAsp were significantly higher in multiple sclerosis patients (*p* < 0.001, Mann–Whitney-U). The percentage of modification was determined by the ion intensities of (Modified YLATASTMDHAR)/(YLATASTMDHAR + YLATAST**M**DHAR) × 100. Controls *n* = 10; multiple sclerosis patients *n* = 8

As noted, a number of tryptic peptides contained more than one PTM. Two such sites involving Asp145/Gln147 and Asp 48/Arg 49 were depicted in Figs. 5 and 6. Evidence for another such interplay was found for Met21 and Asp22 (^14^YLATASTMDHAR^25^). Significant oxidation of Met21 was detected in all control MBP samples (Fig. 7a). Since Met can oxidise artifactually during extraction or digestion [24], and no precautions were taken specifically to minimize oxidation, this PTM was initially disregarded. However, other considerations suggest that Met oxidation in MBP from controls may be real. Firstly, very little oxidation of Met21 was found in MBP from MS patients treated in exactly the same manner. Secondly, in controls, the levels increased as a function of age. Thirdly, when Met sulfoxide levels were monitored in the same tryptic peptide where racemization of Asp22 was also present (Fig. 7b), a similar pattern of Met sulfoxide formation was noted. In this case, as was found with the L-Asp isoform (Fig. 7a), oxidation of Met 21 increased linearly with age in control MBP and again minimal oxidation was detected in MS patients. IsoAsp22 levels were consistently higher in this Met peptide from MS patients (Fig. 7c). Other authors have described oxidation of Met 21 in MBP with speculation that it may be linked to nearby phosphorylation of Ser or Thr [22].

### MS specific sites of MBP modification

**Fig. 8:**
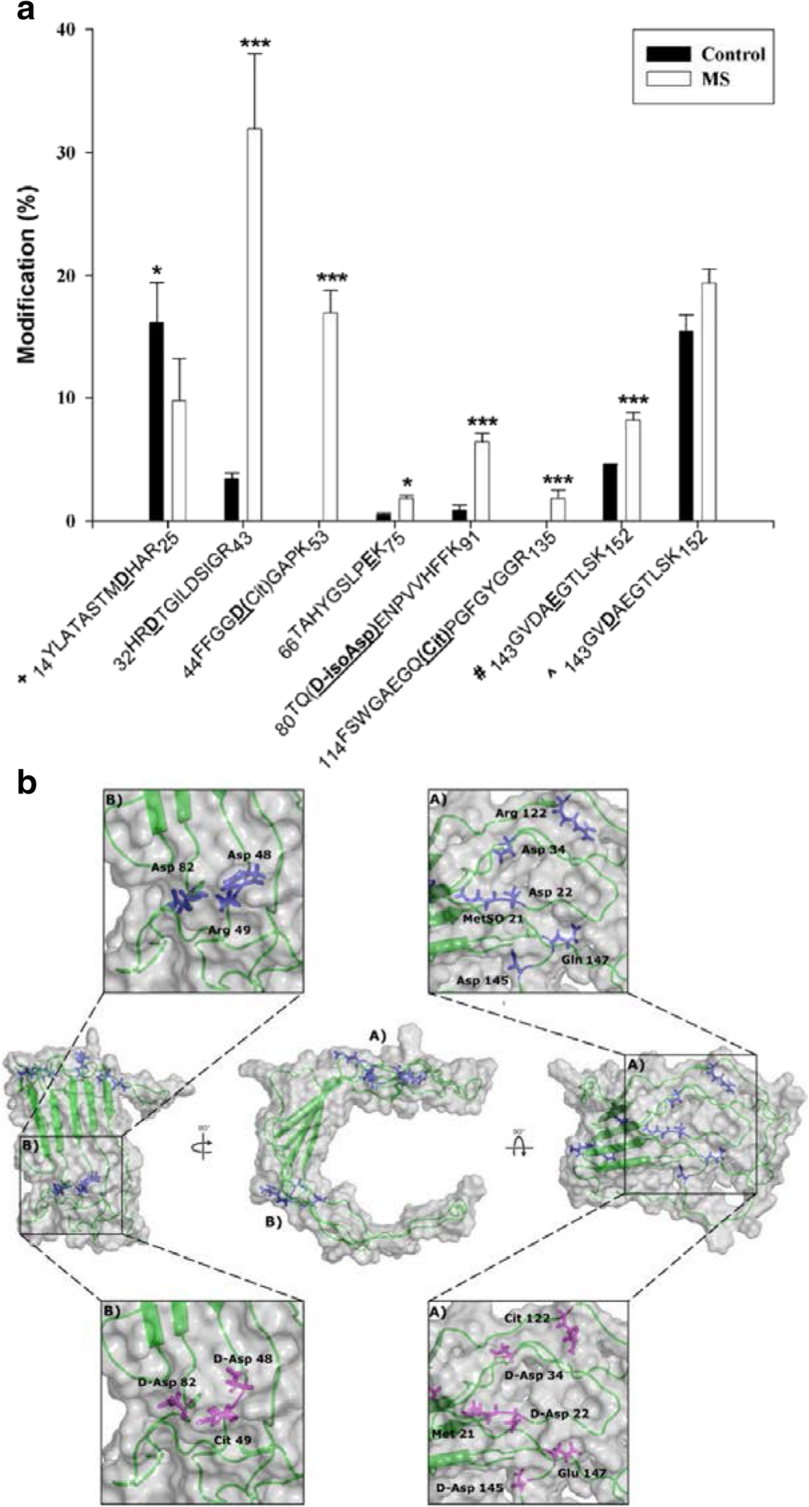
A summary of the sites of modification detected in human MBP. **a** A histogram of the percentage modification at particular sites for controls (**■**) and multiple sclerosis (MS) patients (**□**). The underlined residues in bold correspond to the site of modification in MBP. (+) total Asp racemization in peptide YLATASTM**D**HAR with and without Met oxidation; (#) total deamidation of Gln147 incorporating all isomers of Asp GVDA*E*GTLSK. [^] total Asp145 racemization incorporating the Gln and Glu versions of peptide GV**D**AEGTLSK. Values for Met sulfoxide 21 were not plotted since the levels increased significantly with age. All values are the mean of all ages ± SEM. Asterisks represent level of significance (* *p* ≤ 0.05, ** ≤ 0.01 and *** ≤ 0.001, Mann–Whitney-U). **b** A model of MBP^23^ highlighting the residues that differ significantly in multiple sclerosis. The amino acid residues in blue correspond to the unmodified conformation, those in magenta illustrate the changes in conformation in multiple sclerosis. With the exception of TAHYGSLPQK, all the modifications are clustered within two zones as illustrated; site A) contains six and site B) contains three modified residues. At each of these amino acid residues, the extent of modification was found to be significantly different in multiple sclerosis patients compared with controls. Sites of Asp racemization labeled as D-Asp in 6b, include all Asp isomers (i.e. D-Asp, L-isoAsp and D-isoAsp). In the case of TQDENPVVHFFK only the D-isoAsp version was significantly different

A summary of MBP sites where PTMs differed significantly in controls and MS patients is shown in Fig. 8a. Mapping of these sites onto a proposed structure of MBP [3] (Fig. 8b) showed that these sites were clustered into two distinct regions. Site A incorporated the most abundant site of racemization in MS patients (Asp34) with an estimated 32 % of this residue racemized. Site B contained a dual modification; racemization at Asp48 together with citrulline 49. Asp48 and citrulline 49 were present in each of the MS patients but were not detected in controls. This intriguing finding may suggest that PTMs within two exposed patches of MBP could be involved in provoking an immune response that ultimately results in MS.

## Discussion

This study has revealed the diversity and extent of modifications present in MBP from the normal adult human brain. In addition, MBP from MS patients displayed several sites where the covalent alteration differed significantly from that of normal individuals.

Racemization was found to be a widespread PTM of MBP, with some sites present specifically in MS patients. In particular racemization of L-Asp to the three other Asp isomers (see Fig. 2) was an abundant modification and conversion of L-Asp to isoAsp was characterized at several sites. One particular PTM; isoAsp48 in combination with citrulline 49 was detected only in MS patients (Fig. 6a).

At other sites, isoAsp was present in MS patients at levels significantly higher than those of the controls (Asp34 and Asp82). Although MBP has been investigated for PTMs such as citrullination, phosphorylation, methylation and deamidation e.g. [16, 22], age-related and MS-related changes have not been previously reported. Analysis of human MBP for MS-related modifications in this study, show that it is essential to evaluate such PTMs in relation to the background of age-related changes.

Formation of isoAsp at several sites is likely to be significant in terms of the conversion of MBP to a novel antigenic form that could potentially act to trigger an immune response. This is because others have shown that replacement of L-Asp by an isoAsp in a peptide converts it to an immunogen [9, 29]. If, as in the case of MBP where isoAsp 48 is adjacent to another known immunogenic amino acid, citrulline (49), then this site may be particularly antigenic. This dual modification was detected only in MS patients (Fig. 8). It should however be noted that we cannot definitively conclude that the MS -specific sites of modification detected in this study are the cause of MS; their formation may be a consequence of the disease. This is currently a subject of further investigations.

The human body contains a number of long-lived proteins and their degradation may contribute to age-related diseases [36–38]. Rodent studies using a diet of labelled amino acids showed that MBP was a stable protein [35]. In the case of human MBP, the amino acid racemization data alone (Fig. 1) suggest that it, like lens proteins, is a life-long protein [26]. The protein data correlate with recent cellular data [43]. For example, the final number of oligodendrocytes in the human brain is attained by age ~9 and, once formed, they undergo little turn over. In addition mature oligodendrocytes myelinate axons very poorly [40]. Thus any turnover of carbon in myelin that may be associated with an increase in white matter volume [6] appears to involve changes in lipid, while the myelin proteins are retained.

PTMs such as deamidation and racemization documented for MBP (Fig. 8) are consistent with those expected for susceptible amino acids in proteins that reside for years in the body. Due to the sheer number of modifications of different types, the structure of MBP will inevitably be altered in adult myelin compared with that when it was first synthesized. For example, deimination alters the net charge on MBP and this will reduce its binding to the negatively-charged head groups of phospholipids. This could lead to localized disruption of myelin [16]. Deamidation, even at just one site, can lead to significant protein denaturation [12] and racemization of amino acids is also likely to lead to unfolding [17]. In this study we found a number of sites of racemization. With regard to the impact of extensive PTMs on conformation, it should be emphasized that the MBP structure shown in Fig. 8b is a model [3]. The majority of MBP is unstructured and this accords with the fact that PTMs, such as racemization, identified here are typically localized to unstructured regions [19, 20, 41].

If PTMs of MBP are indeed responsible for inducing MS, their age-dependent profile can account for an otherwise puzzling observation i.e. that MS often begins in the fourth decade of life. It is clear from the graphs (Figs. 3, 4, 5a, 6 and 7) that by the age of 30, MBP has undergone a plethora of PTMs. Every amino acid change effectively introduces a “non-self” motif into the protein; thus each one, or a combination of several, could potentially generate an immune response. There are precedents for amino acid racemization, in particular isoAsp formation, eliciting an immune reaction, e.g. autoimmunity to histone H2B in systemic lupus erythematosus [8, 10].

There is still much to be understood about the detailed molecular architecture of myelin and questions remain to be answered in relation to the part played by MBP. In relation to this, if MBP is an intracellular protein, how could it act as a trigger for an immune response? The literature is not clear as to whether all of MBP is indeed intracellular. In addition, if indeed all of MBP were originally intracellular e.g. in childhood, it is also possible that changes that occur with age could lead to partial myelin breakdown and therefore exposure of MBP to the immune system. Previous research has shown that MBP fragments can be presented by MHC activating CD8+ cells [44], and it is well known that in mouse models of MS, demyelination can be induced by injection of MBP fragments [1]. A number of studies support a role for MBP in the progression of the MS [5, 31, 32].

Since the brains of all people contain MBP that is highly modified by age 20, it is conceivable that the reason some people develop MS, while others do not, can be traced either to the specific types of PTMs and/or the way a subsequent immune response is modulated. This latter aspect could involve suppression of the immune system and components that modulate it, such as vitamin D, or it might include the masking of altered sites on MBP; for example, chaperones could act to minimize T-cell responses. In this regard, αB-crystallin expression is increased in MS lesions [2]. Given that MBP is highly modified by early adulthood, it will be important in the future to investigate how such a newly generated ‘non-self‘protein is prevented from eliciting an antigenic response in controls. It is likely that other major myelin proteins like proteolipid protein and myelin oligodendrocyte glycoprotein will be modified with age, since these are also long-lived [35, 36] and have also been implicated in MS [14, 15]. Detailed proteomic analysis of these proteins may therefore yield other potential MS-related epitopes.

Our proteomic data revealed specific sites of modification in MBP that were common to all MS patients. One conclusion is that these sites may be particularly antigenic. Other PTM sites have been reported previously [22], e.g. an increase in methylation of Arg107, deimination of Arg at several sites, and a reduction of phosphorylation in MS [22]. The majority of sites of Arg deamination in our study match those reported previously [22]. Deimination at some sites was age dependent (Fig. 6a), but in most cases the amount of citrulline in MBP from MS patients did not differ significantly from control MBP Sites of racemization are generated by spontaneous processes which occur more rapidly in unstructured regions of a protein. For MS patients to display elevated D-isoAsp in some locations suggests that MBP may exist in a different conformation in diseased myelin.

In a recent review [28], Mahad and colleagues considered that MS could be viewed either as a classic autoimmune disease (the so called “outside-in hypothesis”) or as a disease triggered by a foreign, e.g., viral antigen (the so called “inside-out hypothesis”). Our observations provide a means to meld these apparently separate mechanisms into one. The formation of MS-specific PTMs of MBP via spontaneous decomposition mechanisms may effectively convert an abundant neural protein into a ‘non-self antigen’. The recent report of a lymphatic drainage system in the CNS [25] opens up new possibilities of how non-self antigens may be detected by the immune system.

## Conclusions

The finding herein that specific sites of PTM in MS patients are localized in two zones of MBP suggests that these regions may be involved in antigen recognition by the body’s immune surveillance machinery. This discovery unlocks the possibility of selectively masking such sites on MBP using small molecules. If this hypothesis can be verified, it may lead to the development of a new class of drugs that could potentially inhibit the onset of MS, as well as help in modulating the immune response of patients who already have developed the disease.

## Additional file

Additional file 1: Sites of Asp, Asn, Ser and Gln deamidation / racemisation. In addition to the sites of modification described, other sites of modification were detected in MBP. Some differences between MS patients and controls for Asp, Asn, Ser and Gln are summarised. (DOCX 1.84 mb)
