# Supplementary material for: Isoaspartic acid is present at specific sites in myelin basic protein from multiple sclerosis patients: could this represent a trigger for disease onset?
Source: Acta Neuropathol Commun. 2016 Aug 12;4:83. doi: 10.1186/s40478-016-0348-x (PMC4983062; doi:10.1186/s40478-016-0348-x)
Supplement: Additional file 1: — Sites of Asp, Asn, Ser and Gln deamidation / racemisation. In addition to the sites of modification described, other sites of modification were detected in MBP. Some differences between MS patients and controls for Asp, Asn, Ser and Gln are summarised. (DOCX 1.84 mb) [file 40478_2016_348_MOESM1_ESM.docx]

**Additional file 1**

**Aspartic acid**

Asp isomerization was also observed at other MBP sites aside from Asp22, 34 and 145. For example, L-Asp48 and L-Asp82 also showed significant conversion to the other isomers. D-isoAsp 82 (^79^TQ**D**ENPVVHFFK^91^) levels were higher in MS patients (6.4% +/-0.71) than controls (0.85% +/- 0.46) (p < 0.001, Mann-Whitney-U). Asp 48 (^44^FFGG**D**R^49^) showed no significant differences between MS patients (27.4% +/-2.90) and controls (28.2% +/- 2.10) (p = 0.859 Mann-Whitney-U).

**Asparagine**

MBP contains two Asn residues, ^92^**N**IVTPR^97^and ^79^TQDE**N**PVVHFFK^91^. **N**IVTPR showed an age-related change in deamidation increasing from ~1% at age 18 to ~4% by age 78. There were no significant differences between MS patients and controls (p =0.375, Mann-Whitney-U).

**Serine**

Some Ser residues are also racemised in adult MBP (Fig 1c) and this PTM has been described in other long-lived proteins [^1^](#_ENREF_1)^,^[^2^](#_ENREF_2), however D-Ser was not investigated in detail in this study, since it presents some difficulties in analysis. One site, Ser 71 (^66^TAHYG**S**LPQK^75^), was racemised based on co-elution of the D-Ser version of the tryptic peptide and MS/MS analysis, although the extent of racemization did not differ significantly between controls and MS patients.

**Glutamine**

MBP contains six Gln sites aside from Gln147 of which only Gln 73 (TAHYGSLP**E**K 1.04% +/- 0.15 controls, 1.83% +/- 0.23 MS, P = 0.026 Mann-Whitney-U) showed significant differences in deamidation between MS patients and controls. The other five sites either did not show any significant differences or were not detected due to small size of the tryptic peptide (e.g. at the N-terminus).

**Table S1:** Demographics of the brain donors used as controls. PMI, Post mortem interval, LC/MS, LC/MS, Liquid chromatography/mass spectrometry: AAA, amino acid analysis

| **Age (y)** | **Gender** | **PMI (h)** | **Brain pH** | **Cause of death - category** | **Cause of death - clinical** | **Tissue used**  **(LC/MS or AAA)** |
| --- | --- | --- | --- | --- | --- | --- |
| 18 | Male | 28.5 | 6.7 | Cardiac | Primary cardiac arrhythmia | LC/MS |
| 22 | Male | 50 | 6.86 | Trauma | Blunt trauma | LC/MS, AAA |
| 24 | Male | 43 | 6.27 | Cardiac | Undetermined (but consistent with idiopathic cardiac arrhythmia) | LC/MS |
| 33 | Female | 24 | 6.77 | Cardiac | Cardiac arrhythmia; myocardial fibrosis | LC/MS, AAA |
| 37 | Male | 14.5 | 6.46 | Cardiac | Presumed Cardiac Dysrhythmia due to natural cause | AAA |
| 40 | Male | 27 | 6.79 | Vascular | 1.a) Pulmonary thromboemboli b) deep venous thrombosis | LC/MS, AAA |
| 47 | Male | 27 | 6.66 | Cardiac | Ischaemic heart disease. | LC/MS |
| 48 | Male | 17 | 6.62 | Cardiac | Ischaemic heart disease. Coronary artery atheroma | AAA |
| 49 | Male | 38 | 6.92 | Cardiac | Coronary Artery Disease | AAA |
| 51 | Male | 35 | 7 | Cardiac | Cardiomegaly | AAA |
| 57 | Male | 18 | 6.39 | Cardiac | Myocardial Infarction | LC/MS |
| 58 | Male | 39 | 6.49 | Cardiac | Ischaemic heart disease. | AAA |
| 62 | Female | 35 | 6.06 | Cardiac | Hypertensive and atherosclerotic heart disease | AAA |
| 66 | Male | 32 | 6.66 | Cardiac | Cardiomegaly | LC/MS |
| 67 | Male | 25 | 6.7 | Cardiac | Hypertensive Heart disease | AAA |
| 69 | Female | 39 | 6.72 | Cardiac | Coronary Artery Disease and asthma | AAA |
| 73 | Female | 45 | 6.86 | Cardiac | Atherosclerotic cardiovascular disease. | LC/MS |
| 74 | Female | 20 | 6.59 | Cancer | Cancer of breast, liver and bone metastases | AAA |
| 78 | Female | 45 | 6.05 | Toxicity | Multiple drug toxicity (7-amino nitrazepam, nitrazepam and dextropropoxyphene) | LC/MS |
| 81 | Male | 29 | 6.57 | Cardiac | Heart Failure | AAA |
| 87 | Female | 5 | 6.38 | Cancer | Metastatic breast cancer | AAA |
| 98 | Female | 6 | 6.7 | Respiratory | Pneumonia; congestive cardiac failure | AAA |
|  |  |  |  |  |  |  |

**Table S2:** Demographics of MS brain donors. PMI, post-mortem interval. SPMS, Secondary Progressive multiple Sclerosis, PPMS, Primary-progressive multiple sclerosis, RRMS, Relapsing-remitting multiple sclerosis.

| **Age (y)** | **Gender** | **PMI (h)** | **Brain pH** | **Cause of death - category** | **Type of MS** | **Age at onset** |
| --- | --- | --- | --- | --- | --- | --- |
| 65 | Male | 41 | 6.36 | Cardiac | SPMS | 24 |
| 68 | Female | 15.5 | 6.66 | Cardiac | SPMS | 39 |
| 62 | Female | 11 | 6.32 | Vascular | PPMS | 54 |
| 48 | Male | 29 | 6.75 | Cardiac | SPMS | 30 |
| 70 | Male | 21 | 6.17 | Cardiac | RRMS | 28 |
| 36 | Female | 24 | 6.45 | Cardiac | PPMS | 24 |
| 72 | Female | 31 | 6.69 | Cardiac | RRMS | 53 |
| 60 | Female | 14 | 6.73 | Cardiac | SPMS | 27 |
|  |  |  |  |  |  |  |
|  |  |  |  |  |  |  |

**Table S3:** Sites of modification in MBP from control and MS patients. (MS, n= 8; control, n = 10). The numbers in parentheses show the number of samples in which that modification was detected.

|  | **Control** | | **MS** |
| --- | --- | --- | --- |
| Deamidation | **Q74 (7), Q81 (2), N84 (1), Q103 (4), Q147 (10)** | | **Q74 (8), Q81 (3), Q103 (2) Q121 (2), Q147 (8)** |
| Oxidation | **M21 (10), M167 (10)** | | **M21 (8), M167 (8)** |
| Methylation | **R49 (4), R107(6)** | | **R49 (5), R107(4)** |
| Dimethylation | **R49(4), R107(6)** | | **R49 (4), R107(6)** |
| Citrulline | **R25 (5), R31 (2) R33 (3), R49 (10), R65 (4), R97 (10), R107 (9), R130 (5), R158 (10), R162 (10), R169 (9), R170 (9)** | | **R25 (7), R31 (5), R33 (6), R44(1), R49 (8), R65 (5), R78 (2), R97 (6), R107 (7), R122 (6), R130 (6), R158 (8), R162 (8), R169 (8), R170 (8)** |
| Phosphorylation | | **T97 (6), S115 (1)** | **S19(4), T20(2), S71(1), T97 (2), S115 (4),** |





**Figure S1** Deimination of Arg 65 and Arg 122 in MBP with age. a) Tryptic peptide DSHHPA**R**TAHYGSLPQK from controls (●) and MS patients (○). Elevated levels of deimination were found in MS patients (p = 0.01, Mann-Whitney-U). b) Tryptic peptide FSWGAEQ**R**PGFGGYGGR from controls (●) and MS patients (○). Elevated levels of deimination were found in MS patients (*p* < 0.001, Mann-Whitney-U). The percentage of modification was determined by the ion intensities of (deiminated)/ (deiminated + non-deiminated). controls n=10, MS patients n=8


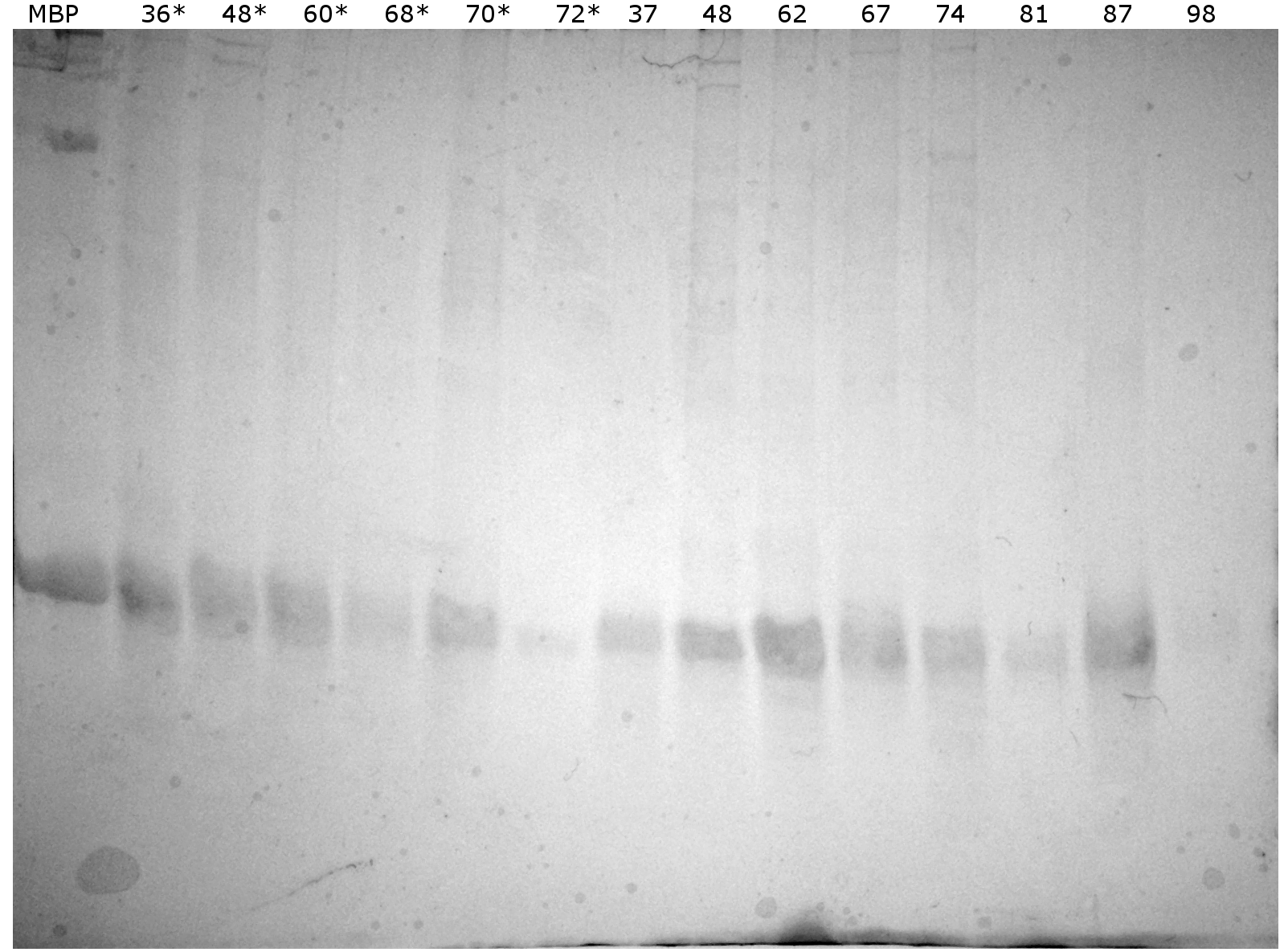


**Figure S2** A representative SDS gel of MBP isolated from the cerebellum as described. MBP refers to a MBP standard, an asterisk indicates MS samples

1. Kaneko, I., Morimoto, K. & Kubo, T. Drastic neuronal loss in vivo by β-amyloid racemized at Ser26 residue: conversion of non-toxic [D-Ser26]β-amyloid 1–40 to toxic and proteinase-resistant fragments. *Neuroscience* **104**, 1003-1011 (2001).

2. Hooi, M.Y.S., Raftery, M.J. & Truscott, R.J.W. Age-dependent racemization of serine residues in a human chaperone protein. *Protein Sci.* **22**, 93-100 (2013).
